# Multiepitope Proteins for the Differential Detection of IgG Antibodies against RBD of the Spike Protein and Non-RBD Regions of SARS-CoV-2

**DOI:** 10.3390/vaccines9090986

**Published:** 2021-09-03

**Authors:** Larissa R. Gomes, Andressa M. Durans, Paloma Napoleão-Pêgo, Jessica A. Waterman, Mariana S. Freitas, Nathalia B. R. De Sá, Lilian V. Pereira, Jéssica S. Furtado, Romário G. Aquino, Mario C. R. Machado, Natalia Fintelman-Rodrigues, Thiago M. L. Souza, Carlos M. Morel, David W. Provance, Salvatore G. De-Simone

**Affiliations:** 1 FIOCRUZ, Center of Technological Development in Health (CDTS)/National Institute of Science and Technology for Innovation on Neglected Population Diseases (INCT-IDPN), Rio de Janeiro 21040-900, Brazil; larissa.gomes@cdts.fiocruz.br (L.R.G.); andressa.durans@cdts.fiocruz.br (A.M.D.); paloma.pego@cdts.fiocruz.br (P.N.-P.); jessica.waterman@cdts.fiocruz.br (J.A.W.); mariana.freitas@cdts.fiocruz.br (M.S.F.); nataliafintelman@gmail.com (N.F.-R.); tmoreno@cdts.fiocruz.br (T.M.L.S.); carlos.morel@cdts.fiocruz.br (C.M.M.); bill.provance@cdts.fiocruz.br (D.W.P.); 2 Interdisciplinary Medical Research Laboratory, Oswaldo Cruz Institute, FIOCRUZ, Rio de Janeiro 21040-900, Brazil; 3 AIDS & Molecular Immunology Laboratory, Oswaldo Cruz Institute, FIOCRUZ, Rio de Janeiro 21040-900, Brazil; nathalia.ramos@ioc.fiocruz.br; 4 Angra dos Reis Health Department, Angra dos Reis 23906-10, Brazil; lilian.venuto@estacio.br (L.V.P.); epidemioangra@gmail.com (J.S.F.); ssa.entomologia@angra.rj.gov.br (R.G.A.); 5 AngraLab Laboratory, Angra dos Reis 23946-010, Brazil; marioangralab@hotmail.com; 6 Immunopharmacology Laboratory, Oswaldo Cruz Institute, FIOCRUZ, Rio de Janeiro 21040-900, Brazil; 7 Department of Cellular and Molecular Biology, Biology Institute, Federal Fluminense University, Niterói 24020-141, Brazil

**Keywords:** SARS-CoV-2, COVID-19, linear B cell epitopes, serodiagnostic, IgG, coronavirus, de novo design

## Abstract

The COVID-19 pandemic has exposed the extent of global connectivity and collective vulnerability to emerging diseases. From its suspected origins in Wuhan, China, it spread to all corners of the world in a matter of months. The absence of high-performance, rapid diagnostic methods that could identify asymptomatic carriers contributed to its worldwide transmission. Serological tests offer numerous benefits compared to other assay platforms to screen large populations. First-generation assays contain targets that represent proteins from SARS-CoV-2. While they could be quickly produced, each actually has a mixture of specific and non-specific epitopes that vary in their reactivity for antibodies. To generate the next generation of the assay, epitopes were identified in three SARS-Cov-2 proteins (S, N, and Orf3a) by SPOT synthesis analysis. After their similarity to other pathogen sequences was analyzed, 11 epitopes outside of the receptor-binding domain (RBD) of the spike protein that showed high reactivity and uniqueness to the virus. These were incorporated into a ß-barrel protein core to create a highly chimeric protein. Another de novo protein was designed that contained only epitopes in the RBD. In-house ELISAs suggest that both multiepitope proteins can serve as targets for high-performance diagnostic tests. Our approach to bioengineer chimeric proteins is highly amenable to other pathogens and immunological uses.

## 1. Introduction

Emerging infectious diseases are a global public health challenge [1]. Their symptoms are easily associated with known pathogens that, along with the absence of diagnostic tools, can delay recognizing a new agent. Combined with the ease and extent of travel today, there is ample opportunity for the disease to spread worldwide, as seen with COVID-19. First suspected from the outbreak of viral pneumonia cases in residents from the Wuhan region of China [2], the novel disease was determined to be caused by a new coronavirus, severe acute respiratory syndrome coronavirus 2 (SARS-CoV-2) [3]. It has a more extended incubation period than SARS-CoV and MERS-CoV [4], which is 5 days on average with a range of 1–14 days before the onset of symptoms. However, two studies have shown that the transmission of SARS-CoV-2 before the beginning of symptoms can vary by 12.6% [5] and 65% [6], which makes up the majority (~86%) of infected people [7].

SARS-CoV-2 was initially identified in patient samples by sequencing [8], which led to the molecular detection of viral genome RNA as the gold standard for diagnosis [9]. While the detection of viral nucleic acid is a definitive indication of an infection, the infrastructure and personnel requirements, along with the time, cost, and discomfort of sample collection, are prohibitive for its implementation in the surveillance of large populations. Furthermore, studies suggest that false-negative test results can occur in up to 40% of swab and sputum specimens [10]. Serology-based diagnostics offer the distinct advantage that pathogen-specific antibodies are available for detection in any sample of blood.

In addition, serodiagnostics can be performed quickly and economically scaled to screen large populations. A wide range of commercialized tests have been launched based principally on detecting antibodies to SARS-CoV-2 internal nucleoprotein (*n*) or external spike glycoprotein (S). The choice of N and S offered rapid production capabilities as recombinant proteins and leveraged the observations with SARS and MERS that showed these proteins to be immunogenic [11,12,13]. The trade-off was in the specificity and sensitivity of the tests, exquisitely related to the target proteins used to capture pathogen-specific antibodies in the patient sample. More directly, their complement of epitopes that can be bound by antibodies.

Our experience with mapping the epitomes of various viruses has shown that individual viral proteins contain a mixture of epitopes with differences in two characteristics important for serodiagnosis: affinity for capturing antibodies and their uniqueness to the pathogen. Here, we identified linear B cell epitopes in the S, N, and ORF3a proteins that were used to bioengineer a chimeric protein containing a high number of epitopes from outside of the RBD of the S protein (Dx-SARS2-noRBD); achieved by inserting their sequences into the thermal green protein (TGP) to replace the non-ß sheet regions. In addition, another chimeric protein was created using only epitopes in the RBD (Dx-SARS-RBD). Computer modeling suggested that both proteins would retain the ß-barrel structure of TGP with the epitopes on the surface, and each was easily expressed in bacteria. Furthermore, as targets for in-house ELISAs, both displayed excellent performance. The results suggest these two multiepitope proteins could be used in the next generation of SARS-CoV-2 serodiagnostic assays and that our approach could be a model for generating immunological tools for other emerging pathogens.

## 2. Materials and Methods

### 2.1. Patient Samples and Ethical Approval

The Human Research Ethical Committee of the University of Estacio de Sá (CAEE number 33090820.8.0000.5284) approved this study, and volunteers provided written informed consent. An initial sera panel comprised 185 serum samples obtained in two distinct groups: asymptomatic (*n* = 52) and symptomatic (*n* = 133). All were confirmed positive for a SARS-CoV-2 infection by a PCR diagnostic test. Another 205 sera were obtained from individuals with no symptoms or who were PCR negative for a SARS-CoV-2 infection. Additional negative controls included 51 sera collected before the start of the pandemic from healthy individuals as well as patients diagnosed with malaria (*n* = 33), dengue (*n* = 48), and chikungunya (*n* = 13) to evaluate cross-reactivity to pathogens endemic to Rio de Janeiro, Brazil. In addition, a panel of 166 sera was collected from individuals with suspected contact with individuals with COVID-19. RT-PCR on nasopharyngeal or oropharyngeal swabs was used to confirm or eliminate cases of COVID-19. Serum samples from individuals with confirmed cases of malaria, dengue, and chikungunya were collected before the onset of the COVID-19 pandemic and generously provided by the Laboratory of Flavivirus of the Oswaldo Cruz Institute of Fiocruz in Rio de Janeiro and the Laboratory Central of Public Health of the state of Ceará, Brazil. Serum from healthy donors, also collected before the pandemic, was provided by HEMORIO, a centralized network of blood donor facilities of the state of Rio de Janeiro, Brazil. To ensure patient privacy, all samples were provided without identifying information.

### 2.2. B-Linear Epitope Mapping

The complete sequences of N, S, and ORF3a proteins of SARS-CoV-2 were retrieved from GenBank for the first deposited sequence of a patient from Wuhan, China (MN908947.3) through access to the Uniprot database (http://www.uniprot.org/: accessed 27 January 2020). Microarrays of peptides and a pool of human patient sera were used to map linear B cell epitopes using an Auto-Spot Robot ASP-222 (Intavis Bioanalytical Instruments AG, Köln, Germany) according to a previous SPOT synthesis protocol [14,15,16]. Consecutive reactive peptides (Figure S1) were interrogated by their sequence overlap and amino acid composition to define epitopes that were subsequently analyzed by a PIR-peptide match (https://fermi.utmb.edu/; accessed 18 March 2020) and BLAST homology search (https://blast.ncbi.nlm.nih.gov/Blast.cgi; accessed 12 March 2020). Only epitopes that showed exclusivity to SARS-CoV-2 were selected to be used in this work (Table 1).

### 2.3. Gene Synthesis, Protein Expression, and Purification

The amino acid sequences of the epitopes that were determined to be specific for SARS-CoV-2 and highly reactive as peptides were integrated into the sequence of thermal green protein (TGP) in silico to design singular chimeric proteins by replacing some of its non-ß-strand sequences as detailed in Table 1. In some instances, a balancer sequence was included to minimize potential conformational constraints on the epitope from its emergence and return to the core ß-barrel structure. In addition, a 6xHis tag was included on the amino terminus of Dx-SARS-RBD and at the carboxy terminus of Dx-SARS2-noRBD. The resulting chimeric protein sequences were then back-transcribed into a coding nucleic acid sequence and optimized for expression in bacteria by the propriety software of Twist Bioscience (USA) that provided the gene synthesis and plasmid preparation service. The plasmid pET-24a was used as the expression vector. Standard techniques were used to transform the BL21 (DE3) strain of *Escherichia coli.* Multiple colonies (5–10) were evaluated to identify a clone for each multiepitope protein with a high expression level (Supplementary Figure S2). The plasmid was recovered and sequenced to confirm fidelity to the protein design. The size of TGP increased from a theoretical measure of 27.3 kDa to 39.6 kDa for Dx-SARS2-noRBD and 39 kDa for Dx-SARS2-RBD.

For protein production, an overnight culture was transferred to a larger-volume culture and allowed to grow to the end of the exponential phase (OD 0.6–0.8) before induction with isopropyl β-D-1-thiogalactopyranoside (0.8 mM) for 3 h at 37 °C. Cells were collected by centrifugation, resuspended in lysis buffer (300 mM NaCl, 10 mM phosphate, pH 8.0), and sonicated, and inclusion bodies were recovered by centrifugation. Inclusion bodies were washed twice by resuspension in lysis buffer containing 0.5% Triton-X100 and centrifugation before the final pellet was resuspended in lysis buffer with 8M urea and 25 mM imidazole overnight at 4 °C. For purification through the his-tag, the solubilized protein was clarified by centrifugation and applied to a HisTrap™ column (GE Healthcare, USA) using an Aktä prime chromatography system (GE Healthcare). Bound proteins were eluted by an imidazole gradient up to 500 mM. Fractions with Dx-SARS2-RBD or Dx-SARS2-noRBD were confirmed by SDS-PAGE, quantified, and used directly for in-house ELISAs.

### 2.4. Molecular Modeling

Theoretical models of Dx-SARS2-RBD and Dx-SARS2-noRBD were generated on the I-TASSER server (http://zhanglab.ccmb.med.umich.edu/I-TASSER/; accessed 19 February 2020, 23 April 2020, and 8 May 2020), which combines the methods of threading, ab initio modeling, and structural refinement [17,18]. The model for each multiepitope protein with the highest TM score and c-score was chosen for visualization using PyMol (V2.2.3; Schrödinger, LLC, New York, NY, USA).

### 2.5. In-House ELISAs

Immunolon 2HB plates (Immunochemistry Technologies, Bloomington, MN, USA) were used with three washes between each step with phosphate-buffered saline plus 0.05% Tween^®^ 20 (PBS-T). Step 1 coated each well overnight at 4 °C with 200 ng of purified Dx-SARS2-RBD or Dx-SARS2-noRBD in coating buffer (50 mM Carbonate-Bicarbonate Buffer, pH 9.6). Step 2 blocked free binding sites with 2.5% BSA in PBS at 37 °C for 1 h. Step 3 was a 1 h incubation at 37 °C with serum samples diluted 1:100 in PBS-T. All samples were analyzed in duplicate. Step 4 was a 1 h incubation at 37 °C with a biotin-labeled anti-human IgG secondary antibody (1:60,000; Sigma-Aldrich, St. Louis, MO, USA). Step 4 was a 1 h incubation with NeutrAvidin-HRP (1:8000; Thermo Fisher Scientific, Waltham, MA, USA). The last step was the addition of 3,3′,5,5′-tetramethylbenzidine (1-Step™ Ultra TMB-ELISA substrate solution, Thermo Fisher Scientific) for 20 min before the reaction was stopped by adding 1 N sulfuric acid. An ELISA reader (Bio-Tek, Winooski, VT, USA) was used to measure the absorbance at 450 nm. All plates included a blank well wherein PBS was substituted for a patient sample. The blank value was subtracted from the absorbances measured for all other wells before plotting and analysis. The dilution values for the secondary and NeutrAvidin were determined by serial dilution experiments with a pool of highly reactive positive patient sera compared to a pool of serum collected pre-pandemic from healthy individuals.

### 2.6. Statistical Analysis

Data were graphed and analyzed by Prism software (GraphPad version 6, San Diego, CA, USA). A receiver operating characteristic (ROC) curve was created for each multi-epitope protein to determine the cutoff as the absorbance with the best likelihood score or 100% specificity and 100% sensitivity. The D’Agostino and Pearson test and the Kologorov–Smirnv test, followed by Student’s t-test, were used to test the normality of datasets, and the variance homogeneity assumption was confirmed. The ANOVA (Brown–Forsythe and Welch) parametric test was chosen to analyze variance with confidence intervals. The reactivity index (RI) reflected the absorbance divided by the cutoff determined by the ROC analysis of each protein. All results >1.1 were considered positive and <0.90 were deemed to be negative. Samples with an RI value of 1.0% ± 10% were defined as being in a gray zone and deemed inconclusive.

## 3. Results

### 3.1. Bioengineering Multiepitope Proteins Specific for SARS-CoV-2

Two different multiepitope proteins were designed based on the epitopes identified to distinguish between an immune response against SARS-CoV-2 and the generation of potent neutralizing antibodies. To detect neutralizing antibodies, the three epitopes in the RBD of the spike protein were chosen (Table 1). A fourth sequence that was not identified in the SPOT synthesis analysis was also selected. It was present in the RBD region and had a random structure that suggested it could potentially interact with antibodies. The in silico bioengineering of the chimeric protein involved substituting the non-ß sheet regions of the green thermal protein (TGP) with the amino acid sequences of the epitopes. Ultimately, the final protein, referred to herein as Dx-SARS2-RBD, had each epitope sequence represented twice. The second protein, Dx-SARS2-noRBD, included a single copy of each of the 11 epitopes: 4 from the spike protein outside the RBD region, 3 from the nucleoprotein, and 4 from the ORF3a.

**Figure 1:**
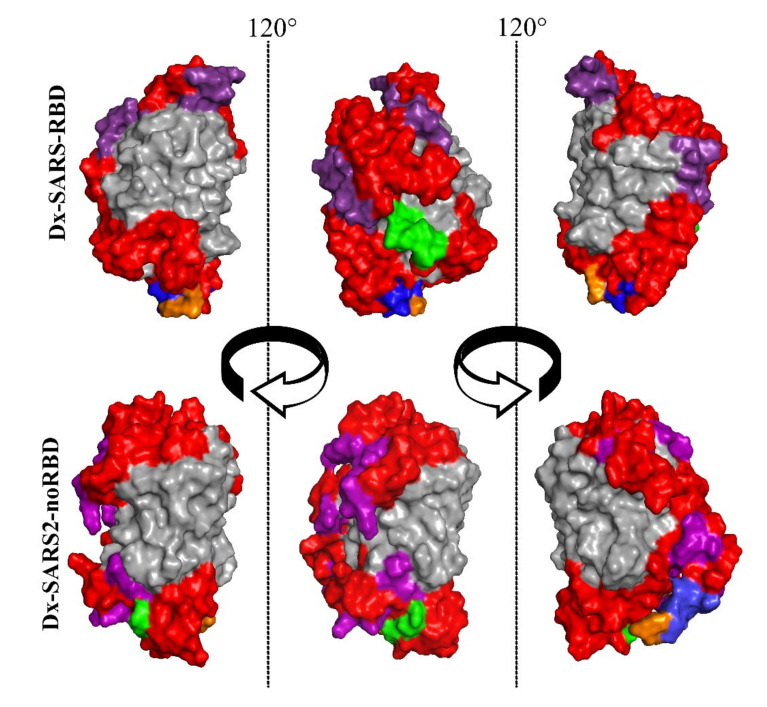
Space-filling models of Dx-SARS-RBD and Dx-SARS2-noRBD multiepitope proteins. The amino acid sequences of Dx-SARS-RBD (**top** row) and Dx-SARS2-noRBD (**bottom** row) were submitted to I-Tasser for computer modeling without a template. The resulting structures were converted into space-filling models using PyMOL and color-coded gray for the receptacle, red for epitopes, purple for spacers, green for the amino terminus, blue for a 6xHis tag, and orange for the carboxy terminus.

Before gene synthesis, Dx-SARS2-RBD and Dx-SARS2-noRBD were modeled on I-Tasser using the crystal structure of TGP (PDB 4TZA; [19]) as a template. The first iteration of Dx-SARS2-RBD returned a configuration wherein one epitope was incorporated into the ß-barrel of TGP. This observation led to the inclusion of sequences to act as balancers expected to minimize stress on forming the ß-barrel structure. It was also anticipated to prevent distortion of the epitope linearity that could negatively influence its antibody interaction. Figure 1 shows the space-filling models with two 120° rotations. The core ß-barrel structure (gray) was preserved in both cases, and the epitopes (red) appeared on the surface. After gene synthesis directly into the pET24 expression vector, both multiepitope proteins were expressed at high levels (>200 µg/mL). While both proteins were present in the soluble fraction, the bulk was in inclusion bodies. The insoluble protein was solubilized in 6M urea and purified by affinity chromatography using an included 6xHis tag for purification.

### 3.2. Performance of Dx-SARS2-RBD and Dx-SARS2-noRBD in Serodiagnosis

**Figure 2:**
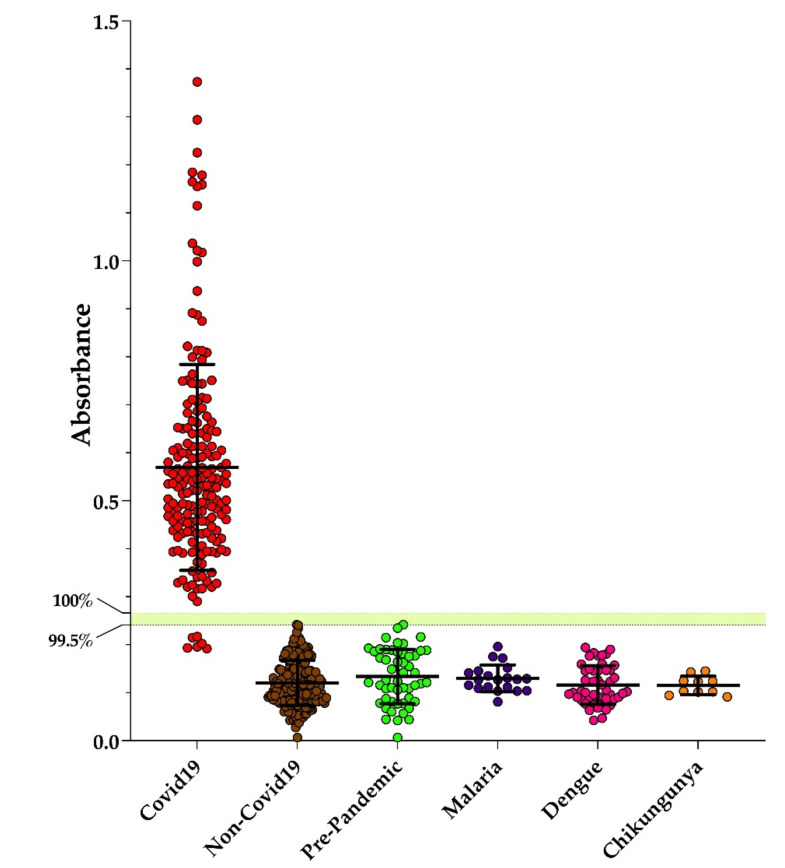
Performance of Dx-SARS2-RBD as a target in an ELISA. ELISA plates were prepared overnight with 200 ng of purified Dx-SARS2-RBD protein before performing an immunoassay to detect human IgG in sera collected from patients confirmed with COVID-19 (*n* = 185). Controls included non-COVID-19 patients (*n* = 205) and sera collected before the start of the pandemic from healthy individuals (pre-pandemic, *n* = 51) as well as patients diagnosed with malaria (*n* = 20) dengue (*n* = 48), and chikungunya (*n* = 10). The median with the SD is shown. The two cutoffs were determined from ROC analysis.

**Figure 3:**
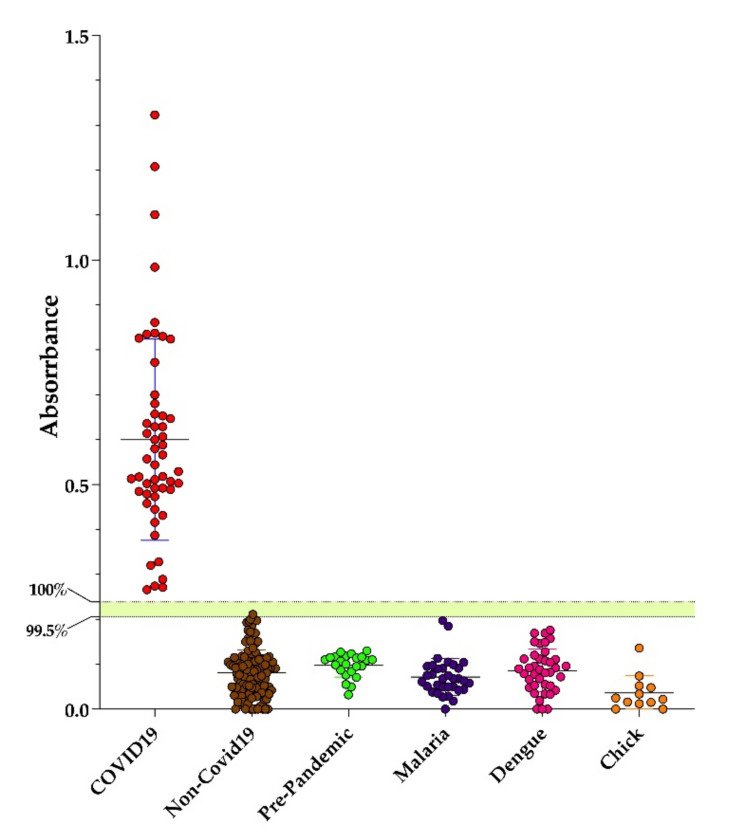
Performance of Dx-SARS2-noRBD as a target in an ELISA. ELISA plates were prepared overnight with 200 ng of purified Dx-SARS2-noRBD protein before performing an immunoassay to detect human IgG in sera collected from patients confirmed with COVID-19 (*n* = 52). Controls included non-COVID-19 patients (*n* = 127) and sera collected before the start of the pandemic from healthy individuals (pre-pandemic, *n* = 23) as well as patients diagnosed with malaria (*n* = 33) dengue (*n* = 39), and chikungunya (*n* = 12). The median with the SD is shown. The two cutoffs were determined from ROC analysis.

The performance of Dx-SARS2-RBD and Dx-SARS2-noRBD in the specific detection of anti-SARS-CoV-2 antibodies was evaluated by in-house ELISAs. After coating ELISA plates with target proteins overnight and blocking, a panel of sera from hospitalized patients that had been independently confirmed with COVID-19 by PCR were tested. Several negative controls were used that included collections both before and after the COVID-19 pandemic. Samples included those from healthy individuals and patients with malaria, dengue, or chikungunya that served to test for cross-reactivity with pathogens endemic to Rio de Janeiro, Brazil. The post-pandemic samples were obtained from individuals who were symptom-free and negative on PCR. As can be seen in Figure 2, Dx-SARS2-RBD was non-reactive with all the negative controls. For the positive samples, six were non-reactive and the rest were reactive. Using a similar panel of serum, Dx-SARS2-noRBD showed that all positive controls were reactive and all negative controls were non-reactive (Figure 3).

The cutoffs designated in Figure 2 and Figure 3 were calculated from the receiver operating characteristics for each (Table 2). The top cutoff value represents 100% specificity and 100% sensitivity. The lower number represents the highest likelihood score that correlated to specificities of 99.51% and 99.21% for Dx-SARS2-RBD and Dx-SARS2-noRBD, respectively. The area under the curve analysis, which refers to the accuracy of the ELISA performance, showed 0.9984 for Dx-SARS2-RBD and 1 for Dx-SARS2-noRBD (Table 3).

### 3.3. Screening of Individuals with Suspected Contact with COVID-19

**Figure 4:**
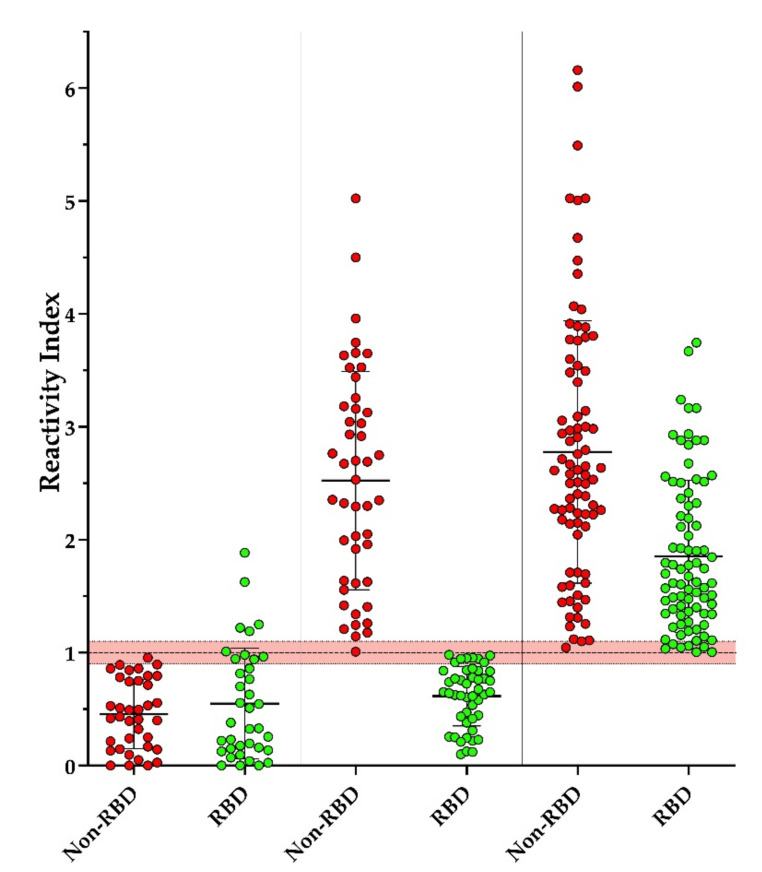
Absence of antibodies toward the RBD in the spike protein of SARS-CoV-2 in a subset of patients positive for anti-SARS-CoV-2 antibodies. Parallel ELISAs were performed using Dx-SARS2-RBD and Dx-SARS2-noRBD proteins as targets for detecting IgG in sera from patients suspected of contact with individuals confirmed with COVID-19 (*n* = 166). A reaction index was calculated using the cutoffs determined from the assays performed in Figure 3 and Figure 4. Percentages were calculated from the total number of patients who were reactive for the RBD (3.7%), non-RBD (35.1%), or both (61.2%). The median with standard deviation is shown.

Our intention was to develop reagents for serological diagnostic tests with improved performance; both multiepitope proteins were used to screen a panel of sera from individuals with suspected contact with persons with COVID-19. As can be seen in Figure 4, three conditions were observed: reactive for Dx-SARS2-RBD and non-reactive for Dx-SARS2-noRBD (3.7%), reactive for Dx-SARS2-noRBD and non-reactive for Dx-SARS2-RBD (35.1%), and reactive for both (61.2%). The reactivity index was employed using the most stringent cutoff of 100% specificity and 100% sensitivity to normalize the data between the two targets that had different cutoffs.

## 4. Discussion

SARS-CoV-2, as an emergent pathogen, was initially detected in central China in December 2019. According to the WHO, the virus quickly spread worldwide within months to infect more than 167 million individuals, resulting in more than 3.4 million deaths (https://covid19.who.int; accessed 26 May 2021). Globally, the incidence of SARS-CoV-2 infections and associated mortality has displayed multiple waves that suggest the approaches employed for containment have been inadequate, attempting to identify infected persons through signs of symptoms, virus detection by molecular techniques, and serological diagnostics. Early diagnosis is crucial for controlling the spread of COVID-19. In addition, the SARS-CoV-2 infection has a reported long-time incubation period, and the risk of virus spread among exposed subjects appears greater than SARS-CoV and MERS-CoV [20].

All ages of the population are susceptible to SARS-CoV-2 infection [21]. However, clinical manifestations differ with age. In general, older men (>60 years old) with comorbidities are more likely to develop a severe respiratory disease that requires hospitalization, whereas most young people and children have only mild infections (non-pneumonia or mild pneumonia) or are asymptomatic [22]. More severe symptoms develop with a median time of 8 days from illness onset. However, the similarities in the presentations of different viral diseases complicate discerning the underlying pathogen, leading to false positives. For SARS-CoV-2, the majority of cases are asymptomatic and will not be detected through clinical symptoms alone.

The gold standard for the earliest detection of a SARS-CoV-2 infection is through a molecular test, RT-PCR [23]. Many commercial kits are available to detect the viral genome targeting the ORF1b, N, E, or S genes. SARS-CoV-2 can be captured by throat swabs, posterior oropharyngeal saliva, nasopharyngeal swabs, sputum, and bronchial fluid, although lower respiratory tract samples have been found to have the highest viral load [24,25,26,27,28,29]. Viruses can also be found in the intestinal tract or blood samples, even when the results are negative using examples from the airways. However, molecular detection can vary due to multiple factors that can lead to rates of false negatives greater than 50% [30]. Recently, the appearance of many variants has raised the possibility of mutations that could interfere with the molecular detection of the virus [31]. Furthermore, when the viral load declines after disease onset, other detection methods are needed to diagnose COVID-19.

Serological tests have the most potential for being implemented at the scale needed to control the current pandemic. With current formats, IgM and IgG antibodies for SARS-CoV-2 are detectable in human serum between 7 and 14 days, respectively, after the onset of symptoms [32,33,34,35,36,37]. While performance can vary depending on other factors such as the viral genotype, comorbidities, immune status, and host genetics [38], the most critical element is the target protein used to bind patient antibodies. The first-generation serological assays have employed recombinant forms of viral proteins. While these targets could be quickly produced, they do not represent the ideal target for specificity and sensitivity.

The immunological characteristics of a target protein can best be described by its complement of epitopes since these are sequences that directly interact with the antibodies to be detected by the assay. In natural proteins, each is a mixture of epitopes that differ in uniqueness to a single pathogen and reactivity for antibodies. Our first step in developing the next generation of serological tests was to map the epitopes in the spike protein, nucleoprotein, and ORF3a of SARS-CoV-2. As expected, a large number were identified, and while this approach does not detect all epitopes, it was not necessary to meet our goal of creating a particular target protein, since it was only essential that an epitope be absolutely unique. After performing a BLAST analysis, 15 were found to be exclusive to SARS-CoV-2.

The major challenge was developing a method to bioengineer a multiepitope coding sequence that would result in a protein [39,40]. To maintain the interactions of the epitopes with antibodies, it was clear that the approach needed to maintain their sequences on the surface of the resulting protein. This eliminated the sequential incorporation of epitopes into a poly-amino acid chain with spacer amino acids since it only provided surface elements and no interior elements. Our solution was to use TGP as a proteinaceous receptacle for the epitope sequences. Years of research have shown that the arrangements of the ß-sheet segments that form the ß-barrel are critical for its generation and characteristics [41,42,43,44]. In contrast, the sequences that connect them are not replaced in low numbers [45,46,47,48]. Therefore, we asked whether a more significant number of these interconnecting regions could be exchanged with the sequences of the SARS-CoV-2 epitopes and still produce a protein that displays immunological characteristics of those epitopes.

We chose to create two different proteins: Dx-SARS2-noRBD for an immune response against SARS-CoV-2 as an indication of an infection and Dx-SARS2-RBD for the RBD on the spike protein, which is highly immunogenic, and antibodies that bind to this domain can be neutralized by blocking the interaction between the virus and its host cell entry receptor, ACE-2 [49,50]. Eleven epitopes were incorporated for DX-SARS2-noRBD that excluded all epitopes in the RBD region. For Dx-SARS2-RBD, three epitopes were duplicated in the multiepitope protein. In addition, another sequence was also included twice due to the presence of ACE-2 interacting amino acids [51].

With TGP as the core to the multiepitope protein, a template was available to perform computer modeling of the candidate proteins. After the first model showed one of the epitopes as a portion of the ß-barrel, balancer sequences were used in some instances. The logic applied was to mirror the size of the epitope with sequences that were expected to form α-helices. This would allow the epitope to be presented fully extended and, with an intervening glycine, avoid any tension on the ends of the ß-barrel. After a couple of iterations, models of the multiepitope proteins were returned that preserved the core structure of TGP (Figure 2). They also showed the epitopes on the surface where they could interact with antibodies. Both proteins were readily expressed in *E. coli*, with the majority found in inclusion bodies.

Importantly, as accessed by in-house ELISAs, their performance suggests that each excellently displays specificity by the absence of cross-reactivity against malaria, dengue, and chikungunya. False-positive results have been reported for dengue, leading to delays in the diagnosis of COVID-19 in endemic areas and a greater spread of the virus [52]. When evaluating their ability to detect antibodies in patients confirmed with COVID-19, Dx-SARS2-noRBD reacted with all patient samples without overlapping with the non-COVID-19 samples. Our attention was drawn to the six samples that were non-reactive with Dx-SARS2-RBD. They were clearly below the cutoff determined by the ROC analysis and formed a group distinct from the rest. Since the proteins were made in series, comparable but not the same serum panels were used in the initial evaluation. After screening the same serum panel from persons with suspected contact with COVID-19, it became clear that there appears to be heterogeneity in the immune response to a SARS-CoV-2 infection.

While most of the patient samples that showed reactivity reacted with Dx-SARS2-noRBD (96.3%), nearly one-third of these did not respond to Dx-SARS2-RBD. Due to the choice of epitopes in Dx-SARS2-RBD, this suggests that some people may not generate neutralizing antibodies against this region of the spike protein in the same manner as the group used to screen the peptide library. The assay would not detect conformationally dependent or post-modification-dependent antibodies against the RBD and does not exclude the existence of other neutralizing sites outside of the RBD. It is not expected that the non-reactive individuals represent any variants of SARS-CoV-2, since the samples were collected well before their discovery. In addition, the mutation N501 found in variants is outside of the epitope mapped here [53,54].

Overall, our results show the importance of determining the epitome of an emergent pathogen to identify those epitopes with the specificity and reactivity needed for biomedical applications. Furthermore, we presented an innovative method to incorporate many epitopes into a chimeric protein that can be applied to current and future diseases. Depending on the desired performance profile of the final protein, different epitopes and sequences can be used for SARS-CoV-2 and COVID-19.

## 5. Conclusions

The devasting impacts of SARS-CoV-2, and its associated disease COVID-19, illustrate the need for innovative approaches to rapidly develop immunological reagents. Here, we aimed to bioengineer two multiepitope proteins that represented the receptor-binding domain of the spike protein involved in neutralization or regions outside of the RBD. First, the epitope maps of the spike protein, nucleoprotein, and ORF3a were determined and showed a unique range of reactivities and subsets. Then, to generate chimeric proteins with the SARS-CoV-2-specific epitopes, the ß-barrel region of the green thermal protein was used as a core protein structure to receive their sequences, which permitted computer modeling before gene synthesis and protein production. In-house ELISAs showed that both proteins display high specificity and sound sensitivity. Furthermore, when used to screen the same patient serum panel, a subset of patients (~1/3) showed no reactivity against the RBD. Overall, the approach described has high potential to improve future serodiagnostics for COVID-19 and can be applied to other current and emerging pathogens.

## 6. Patents

The peptides, proteins, and methodology described in this study are protected under Brazilian and US provisional patent applications BR 10.2019.017792.6 and PCT/BR2020/050341, respectively, filed by FIOCRUZ and may serve as a future source of funding. However, the funding agencies had no role in study design, data collection, analysis, publishing decisions, or manuscript preparation.

## Figures and Tables

**Table 1:** Epitopes, their origin, balancers, and the insertion site in TGP used to engineer Dx-SARS2-RBD and Dx-SARS2-noRBD.

	Epitope	Origin	AAs ^1^	Balancer ^2^	AA in TGP ^3^
**Dx-SARS2-RBD**	FERDISTEIYQAGST	RBD of Spike	464–479	N/A	-NH_2_
	^4^ /GSTPCNGVEGFNCYF	RBD of Spike ^5^	476–491	GSSGEAAKEAAK/	50,58
	NSNNLDSKVGGNYNY	RBD of Spike	437–451	N/A	126,140
	FERDISTEIYQAGST/	RBD of Spike	464–479	/GGSGTSYWKGS	168,172
	GSTPCNGVEGFNCYF	RBD of Spike ^5^	476–491	N/A	184,193
	YFPLQSYGFQPTNGV/	RBD of Spike	490–504	/GSSGEAAKEAAK	204,210
	YFPLQSYGFQPTNGV	RBD of Spike	490–504	N/A	-COOH
	/NSNNLDSKVGGNYNY	RBD of Spike	437–451	GGSGGGASG/ ^6^	-COOH
**Dx-SARS2-noRBD**	LGVYHKNNKSWMESEFRVY/	Spike	141–159	/PAPAP	-NH_2_
	/FIYNKIVDEP	ORF3a	231–240	GGSGEAAK/	38,40
	KNPLLYDANY	ORF3a	136–145	N/A	50,58
	AGNGGDAALALLLLD	Nucleoprotein	221–225	N/A	77,90
	RSYLTPGDSSS/	Spike	246–256	/GGASG	99,102
	ADQLTPTWRV	Spike	625–634	N/A	126,140
	FIYNKIVDEP/	ORF3a	231–240	/GGSGTSYWKGS	168,172
	KNPLLYDANY	ORF3a	136–145	N/A	184,193
	/RPQGLPNNTAS	Nucleoprotein	41–50	GGSGGEAAKG/	204,210
	/LIRQGTDYKHWPQIA	Nucleoprotein	291–305	LAEILQKN/	-COOH
	/GKIADYNYKL	Spike	415–424	GGSGG/ ^5^	-COOH

^1^ Amino acid numbering of the epitope in the protein of origin; ^2^ a sequence included along with the epitope; ^3^ the amino acid numbering for TGP; ^4^ “/” designates the fusion of an epitope with its balancer; ^5^ not discovered by SPOT synthesis mapping; ^6^ a spacer sequence between the two epitopes inserted in the carboxy terminus.

**Table 2:** Cutoffs for ELISAs with Dx-SARS2-RBD and Dx-SARS2-noRBD.

Multiepitope Protein	3× ^1^	Absorbance	Sensitivity (%)	Specificity (%)	Likelihood
Dx-SARS2-RBD	0.36	0.241	100	99.51	198.4
		0.2658	100	100	-
Dx-SARS2-noRBD	0.24	0.2055	100	99.21	127
		0.2385	100	100	-

^1^ Value from the multiplication of the negative control median absorbance by three.

**Table 3:** Analysis of the area under the curve for the ELISAs with Dx-SARS2-RBD and Dx-SARS2-noRBD.

Multiepitope Protein	N (Positive)	N (Negative)	AUC	Std. Error	*p*-Value
Dx-SARS2-RBD	185	205	0.9984	0.0008596	<0.0001
Dx-SARS2-noRBD	52	127	1	0	<0.0001

## Data Availability

The data presented in this study are available on request from the corresponding author.

## Supplementary Materials

The following are available online at https://www.mdpi.com/article/10.3390/vaccines9090986/s1, Figure S1: Plots of signal intensities of peptides from SARS-CoV-2 proteins S, N, and ORF3a synthesized by SPOT-synthesis methodology and revealed with anti-human IgG alkaline phosphatase to the procedure previously described [14,15,16]. Figure S2: SDS-PAGE (10%) analysis of Dx-SARS2-RDB purified by affinity chromatography. Dx-SARS2-RDB within inclusion bodies was solubilized in 10 mM phosphate buffer (pH 8.0) with 8M urea, 300 mM NaCl, and 25 mM imidazole overnight at 4 °C. The sample was clarified by centrifugation (15 k× *g*, 30 min at 4 °C), and the supernatant was applied onto a 1 mL NiTrap column on an Akta system (GE). After exhaustive washing with the same buffer, bound proteins were eluted by a gradient with 8M urea in 10 mM phosphate buffer (pH 8.0) with 500 mM imidazole. Lane 1, load; lane 2, Frxn 1 wash; lane 3, Frx 2 wash; lanes 3–7, Frxns 8–11, respectively (elute); lane 8, Frxn 14. Equal volumes of the sample were loaded. Figure S3: SDS-PAGE (10%) analysis of Dx-SARS2-noRDB purified by affinity chromatography. Dx-SARS2-RDB within inclusion bodies was solubilized in 10 mM phosphate buffer (pH 8.0) with 8M urea, 300 mM NaCl, and 25 mM imidazole overnight at 4 °C. The sample was clarified by centrifugation (15 k× *g*, 30 min at 4 °C), and the supernatant was applied onto a 1 mL NiTrap column on an Akta system (GE). After exhaustive washing with the same buffer, bound proteins were eluted by a gradient with 8M urea in 10 mM phosphate buffer (pH 8.0) with 500 mM imidazole. Lane 1, load; lane 2 Frxn 1 flow-through; lane 3, Frxn 1 wash; lane 3, Frxn 8; lane 4, Frxn 9; lane 5, Frxn 10; lane 6, Frxn 11. Equal volumes of the sample were loaded.
