# Supplementary figures and images for: Multiepitope Proteins for the Differential Detection of IgG Antibodies against RBD of the Spike Protein and Non-RBD Regions of SARS-CoV-2
Source: Vaccines (Basel). 2021 Sep 3;9(9):986. doi: 10.3390/vaccines9090986 (PMC8473315; doi:10.3390/vaccines9090986)

**A**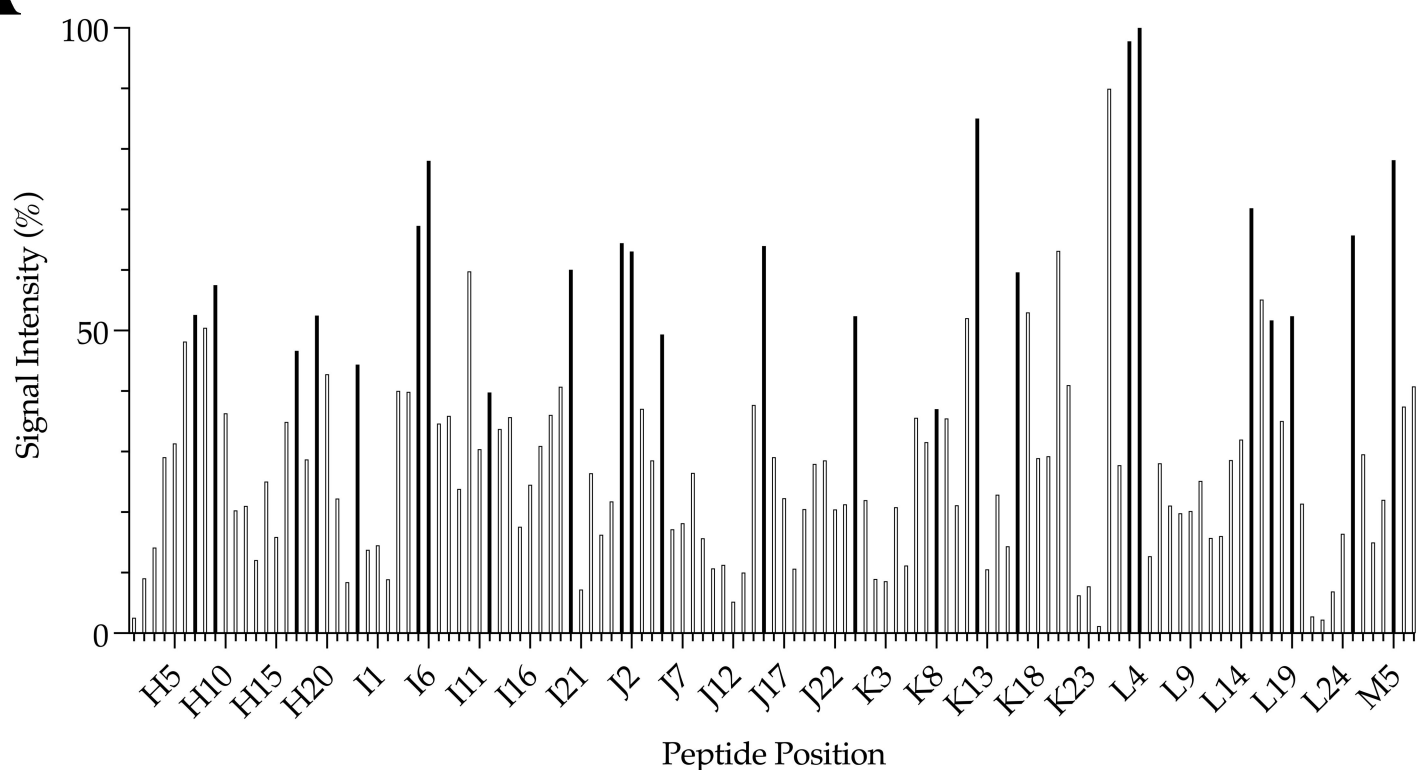**B**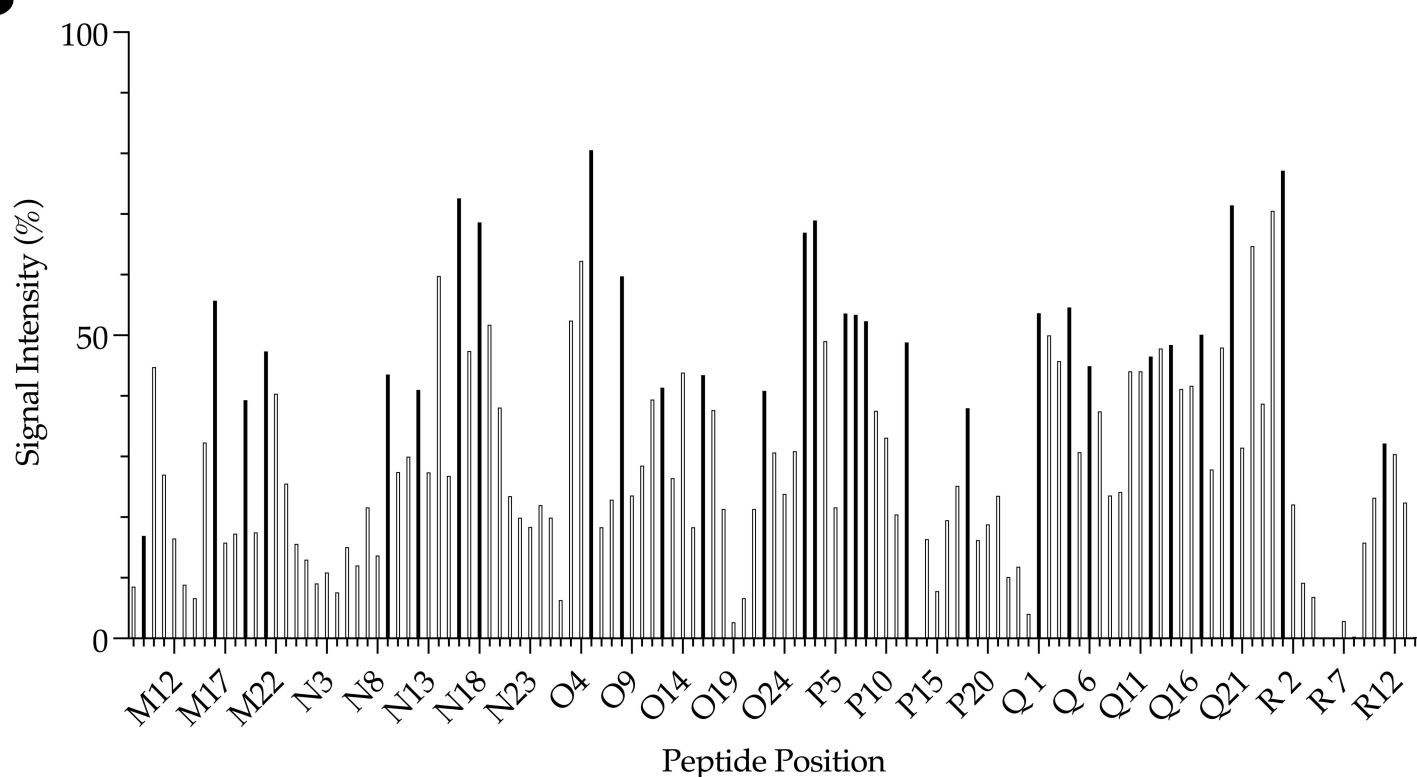**C**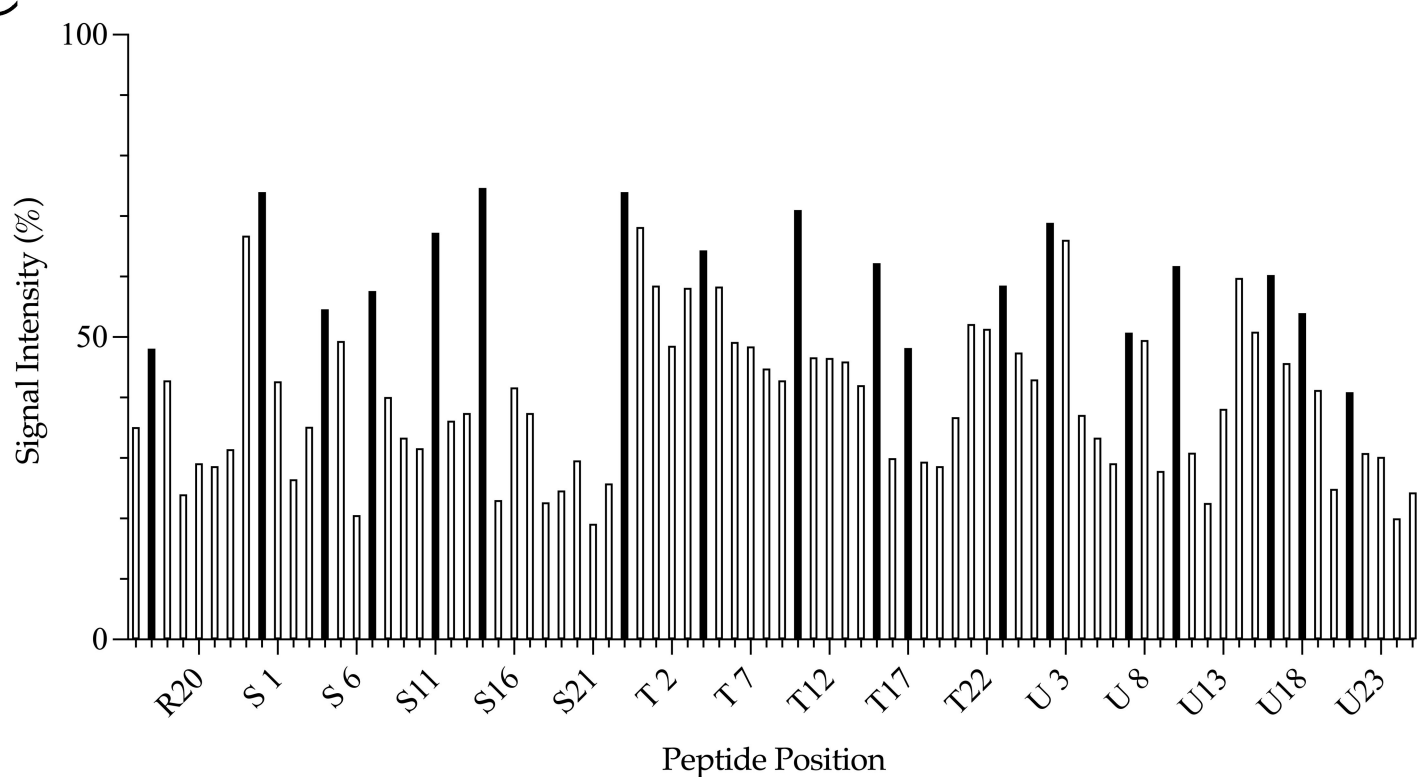**D**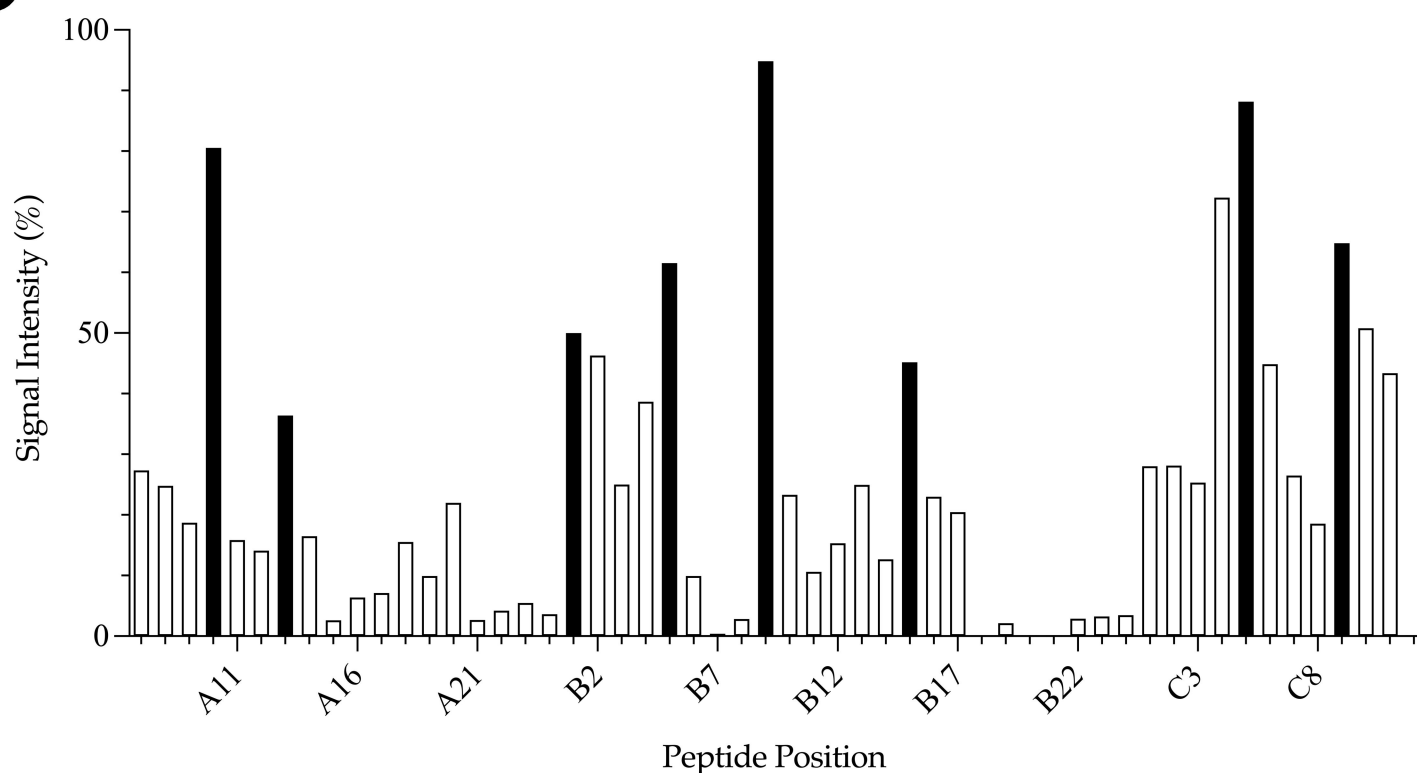

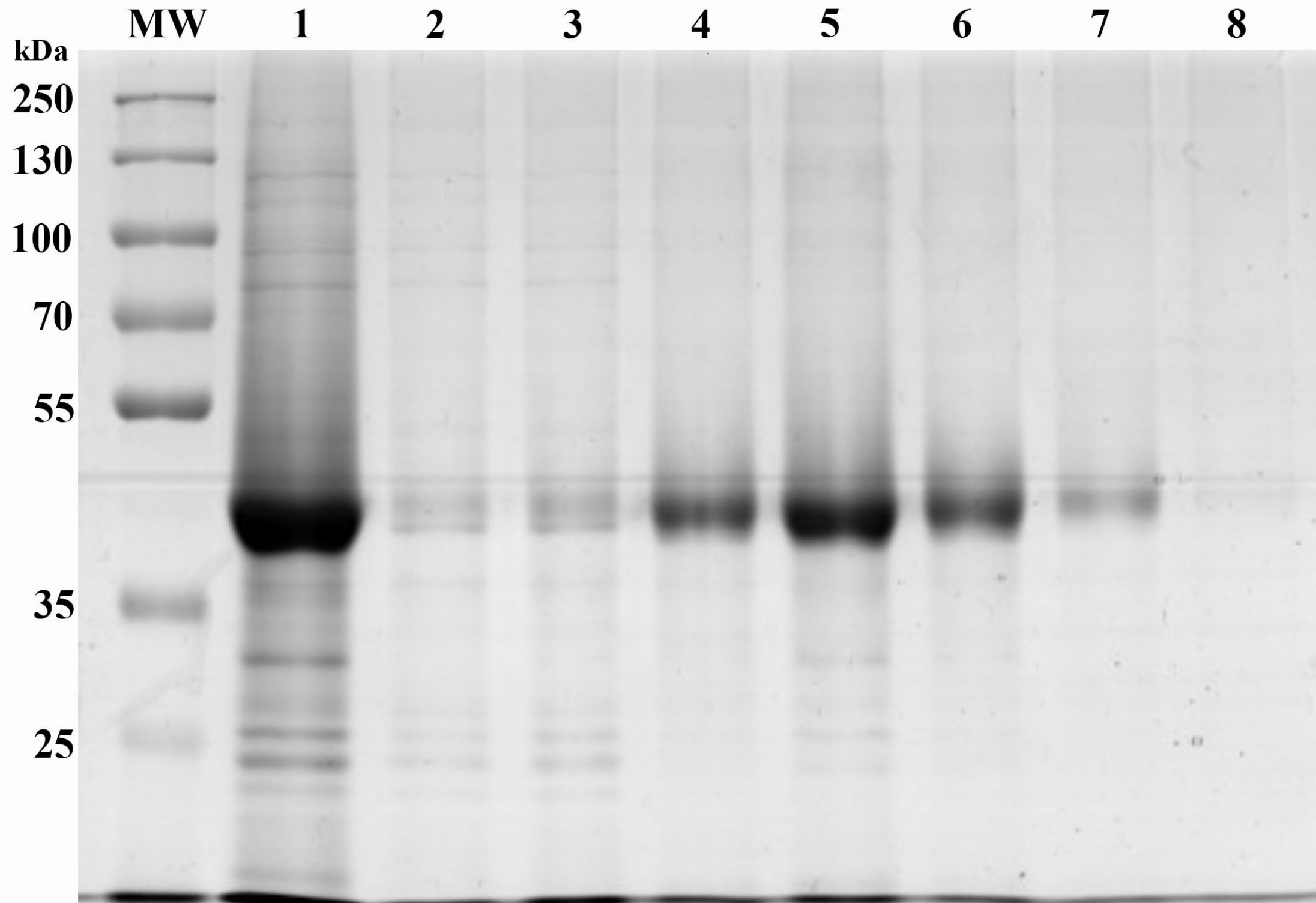

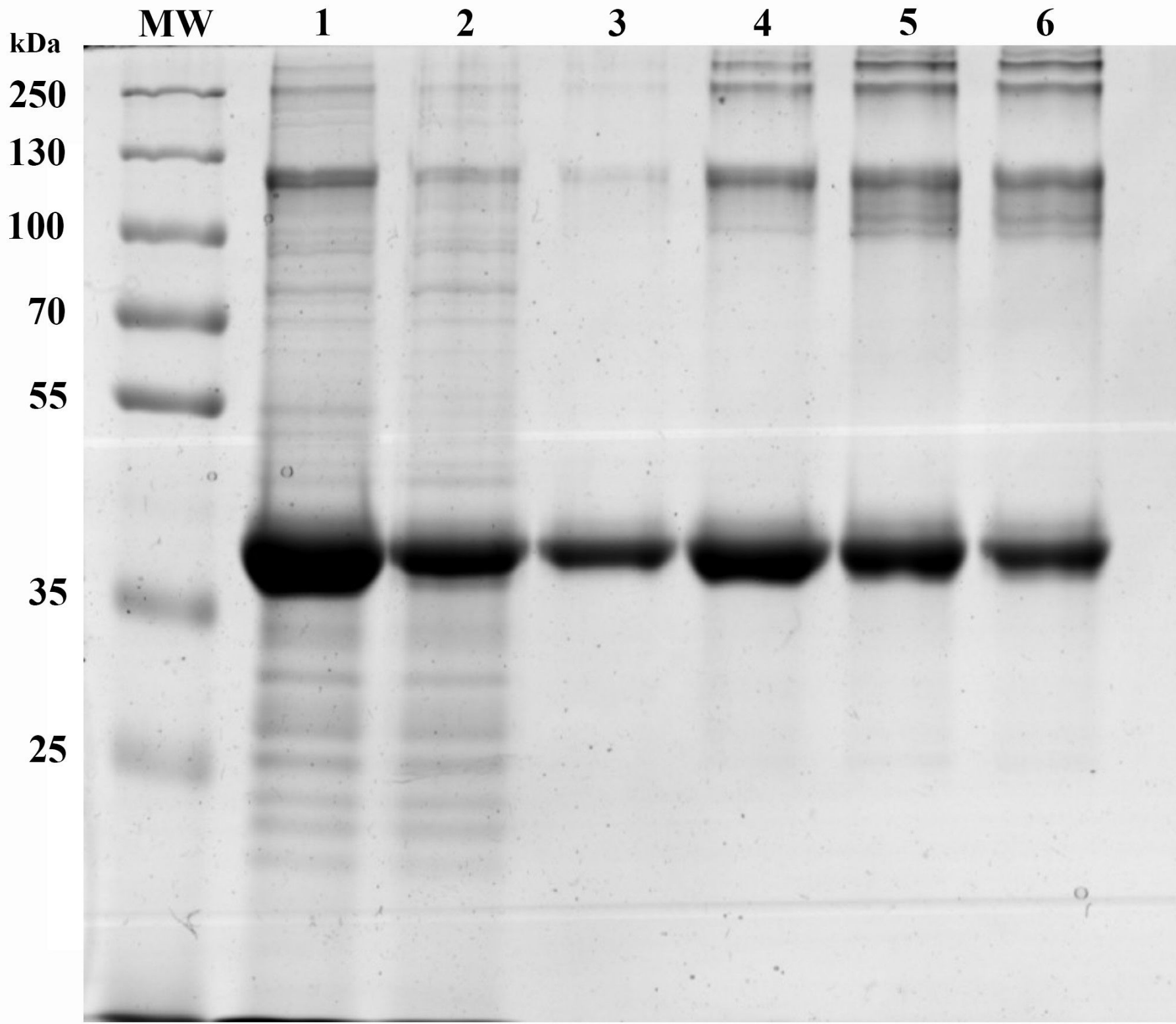

Supplement: Supplementary file 1 [file vaccines-09-00986-s001.zip › vaccines-1286377-supplementary.pdf]
